# Role of glucosamine in the treatment for osteoarthritis

**DOI:** 10.1007/s00296-012-2416-2

**Published:** 2012-03-30

**Authors:** Jean-Yves Reginster, Audrey Neuprez, Marie-Paule Lecart, Nathalie Sarlet, Olivier Bruyere

**Affiliations:** 1 Department of Public Health Sciences, CHU Sart Tilman, University of Liège, Liège, Belgium; 2 Bone and Cartilage Metabolism Research Unit, Department of Physical Medicine and Rehabilitation, CHU Centre Ville, University of Liège, Liège, Belgium; 3 Bone and Cartilage Metabolism Research Unit, Department of Geriatrics, CHU Centre Ville, University of Liège, Liège, Belgium; 4 Bone and Cartilage Metabolism Research Unit, CHU Centre-Ville, Policliniques L. BRULL, Quai Godefroid Kurth 45 (9ème étage), 4020 Liege, Belgium

**Keywords:** Glucosamine, Osteoarthritis, Treatment, Symptoms, Structure

## Abstract

Over the last 20 years, several studies have investigated the ability of glucosamine sulfate to improve the symptoms (pain and function) and to delay the structural progression of osteoarthritis. There is now a large, convergent body of evidence that glucosamine sulfate, given at a daily oral dose of 1,500 mg, is able to significantly reduce the symptoms of osteoarthritis in the lower limbs. This dose of glucosamine sulfate has also been shown, in two independent studies, to prevent the joint space narrowing observed at the femorotibial compartment in patients with mild-to-moderate knee osteoarthritis. This effect also translated into a 50 % reduction in the incidence of osteoarthritis-related surgery of the lower limbs during a 5-year period following the withdrawal of the treatment. Some discrepancies have been described between the results of studies performed with a patent-protected formulation of glucosamine sulfate distributed as a drug and those having used glucosamine preparations purchased from global suppliers, packaged, and sold over-the-counter as nutritional supplements.

## Preclinical research

Glucosamine is an aminosaccharide, acting as a preferred substrate for the biosynthesis of glycosaminoglycan chains and, subsequently, for the production of aggrecan and other proteoglycans of cartilage [1]. Because of the essential role aggrecans play in giving the cartilage its hydrophilicity, compounds enhancing synthesis of aggrecans may be beneficial in cases of OA, a disorder characterized by an increase in matrix structural protein turnover, with catabolism being predominant over synthesis [2].

In vitro, glucosamine sulfate (GS) has been demonstrated to reduce prostaglandin E2 (PGE2) production and interfere with nuclear factor kappa B (NF_B) DNA binding in chondrocytes and synovial cells [3, 4].

Glucosamine inhibits gene expression of OA cartilage in vitro [5]. Long-term oral administration of glucosamine sulfate reduces the destruction of cartilage and upregulation of MMP-3 mRNA in a model of spontaneous osteoarthritis in Harley guinea pigs [6]. Glucosamine can prevent cytokine-induced demethylation of a specific CpG site in the IL1β promoter and this is associated with decreased expression of IL1β [7]. It was suggested that since glucosamine inhibits both anabolic and catabolic genes, the therapeutic effects of glucosamine might be due to anticatabolic activities, rather than due to anabolic activities. GS is a stronger inhibitor of gene expression than glucosamine hydrochloride [8, 9].

## Symptomatic effects in osteoarthritis

Efficacy and safety of GS were tested in several randomized, controlled clinical trials that included patients with OA, predominantly of the knee or spine. In OA of the knee, intramuscular GS (400 mg twice/week for 6 weeks) was compared to a placebo (*n* = 155). At the end of the treatment and 2 weeks after drug discontinuation, a significant difference in the decrease in the Lequesne’s index (an index assessing pain and function and initially developed to identify patients in the need for surgical joint replacement) was observed for the GS group compared to the placebo. A positive rate (responders were those patients with at least a three-point reduction in the Lequesne’s index) was significantly higher in the GS group when considering evaluable patients (55 vs. 33 %) or by intention-to-treat analysis (51 vs. 30 %) [10].

To optimize the long-term compliance of osteoarthritic patients with OA, glucosamine was administered predominantly orally in subsequent clinical trials. In 252 outpatients with OA of the knee [stage I, III], those treated with 1,500 mg/day GS for 4 weeks had a significantly higher decrease in the Lequesne’s index than those receiving a placebo. The response rates were within the same range as those observed with the intramuscular formulation (55 vs. 38 % evaluable patients; 52 vs. 37 % patients in an intention-to-treat analysis) [11]. These results were confirmed by a 16-week, randomized, double-blind placebo-controlled crossover trial of a combination of glucosamine HCl (1,500 mg/day), chondroitin sulfate (1,200 mg/day), and manganese ascorbate (228 mg/day), performed in 34 males from the US Navy diving and special warfare community with chronic pain and radiographic degenerative joint diseases of the knee or low back. While the study did not demonstrate, or exclude, a benefit for the spine, knee OA symptoms were relieved, as evidenced by the changes observed in a summary disease score, incorporating results of pain and functional questionnaire, physical examination score, and running time [12].

In a 3-year trial including 319 patients randomized to 1,500 mg/day of GS or a placebo, preliminary results suggested that GS significantly improved the long-term symptomatic evolution of knee OA assessed by Lequesne’s Algo-Functional index [13]. However, it was observed that glucosamine hydrochloride does not induce symptomatic relief in knee OA to the same extent that GS does. In an 8-week double-blind, placebo-controlled study, followed by 8 weeks off-treatment observation, glucosamine hydrochloride yielded only beneficial results in response to a daily diary pain questionnaire with no effects on the primary endpoint (WOMAC questionnaire) [14]. This questions the importance of sulfate and its contribution to the overall effects of glucosamine.

GS (1,500 mg/day) was also compared to placebo in 162 outpatients with spinal OA (68 with cervical, 57 with lumbar, and 37 with thoracic localizations) and induced a significant improvement of pain and function parameters (visual analog scale) at all localizations. The improvement with glucosamine lasted up to 4 weeks after drug discontinuation [15].

The symptomatic action of GS was also compared to that of nonsteroidal anti-inflammatory drugs. GS (1,500 mg orally) and ibuprofen (1,200 mg) had the same success rate (48 % for GS vs. 52 % for ibuprofen) after 4 weeks in 200 hospitalized patients with OA of the knee. The effect of ibuprofen tended to occur sooner than that of GS (48 % ibuprofen vs. 28 % GS after the first week of treatment). However, significantly fewer patients reported adverse effects (mainly of gastrointestinal origin) with GS (6 %) than with ibuprofen (35 %), and the number of adverse event-related dropouts differed between the two groups (7 % ibuprofen vs. 1 % GS) [16]. These results were perfectly duplicated in another study that included 68 Chinese patients with a nonsignificant difference between ibuprofen and GS (in favor of GS) in the reduction in the symptoms of OA, but GS was better tolerated (6 % of patients with adverse reactions and 0 % of drug-related dropouts) than ibuprofen (16 % of adverse reactions and 0 % of drug-related dropouts) [17]. A total of 319 patients with symptomatic OA of the knee received GS (1,500 mg/day), piroxicam (20 mg/day), both drugs, or a placebo for 12 weeks followed by 8 weeks without treatment. In the GS group, the Lequesne’s index decreased by 4.8 points during treatment, for a decrease of 2.9 and 0.7 points, in the piroxicam and placebo groups, respectively (*p* < 0.001). The association did not differ from GS alone. GS did not differ in safety (14.8 % incidence of adverse events during treatment) from placebo (23.7 %) but was significantly better tolerated than piroxicam (40.9 %) or the association (35 %). The improvement in GS-treated patients persisted during the 8-week follow-up period, whereas the improvement with piroxicam did not [18].

In 45 adult subjects diagnosed with temporomandibular joint (TMJ) OA, GS (1,500 mg/day) and ibuprofen (1,200 mg/day), given for 90 days, both induced significant improvement in TMJ pain with function and pain-free and voluntary maximum mouth opening. Between-groups comparison reveled that patients taking GS have a significant greater decrease in TMJ pain with function and used less acetaminophen (chosen as rescue medication) during the 30-day period following the treatment [19].

Few investigations have tested alternative routes of administration for GS. No head-to-head comparison between the oral and topical routes is currently available. However, a topical application of a preparation containing glucosamine sulfate, chondroitin sulfate, and shark cartilage reduced, within 4 weeks, pain related to knee OA to a significantly greater extend than a placebo cream [20].

Studies with less stringent methodology did not, however, systematically replicate these positive results. In a study of pragmatic design, including 80 patients with a wide range of pain severity from knee OA, the administration of GS (1,500 mg/day for 6 months) did not provide significant pain relief compared to the administration of calcium carbonate (CC). It should be emphasized, however, that the GS preparation used in this trial was an over-the-counter (OTC) formulation containing a mixture of GS, vitamin C, and CC [21]. Similarly, when using another OTC preparation of GS, Rindone et al. [22] were unable to detect an analgesic effect of 1,500 mg of GS daily over 2 months, compared to placebo, in 98 patients with OA of the knee. Both studies were performed with GS preparations purchased from global suppliers and packaged and sold OTC as nutritional supplements. They are not regulated as drugs and might have important variations in content [23, 24]. Noteworthy is that both above-referenced trials [21, 22] were conducted without performing any quality control assays for GS. In a prototypical double-blind, randomized, placebo trial of GS (1,500 mg/day) among subjects recruited and followed entirely over the Internet, no differences between treatment and control groups were observed over 12 weeks concerning pain, stiffness, or function on total WOMAC scores. In this trial, the initial GS (OTC) provider declined to supply placebo capsules during the course of the study and the patients were subsequently treated with a glucosamine HCl formulation, manufactured to pharmaceutical grade purity [25].

A National Institutes of Health sponsored study labelled the glucosamine/chondroitin arthritis intervention trial (GAIT), examined placebo versus glucosamine hydrochloride (500 mg three times daily) versus chondroitin sulfate (400 mg three times daily) versus the combination of glucosamine and chondroitin versus celecoxib (200 mg/day) in a parallel, and blinded 6-month multicentre study of response in knee OA [26]. The primary efficacy variable was a 20 % improvement in knee pain from baseline to 24 weeks. Overall, glucosamine hydrochloride and chondroitin sulfate were not significantly better than placebo in reducing knee pain by 20 %. However, for patients with moderate-to-severe pain at baseline, the rate of response (OMERACT–OARSI criteria) was significantly higher with combined therapy than with placebo (79.2 vs. 54.3 %, *p* = 0.002).

The Glucosamine Unum In Die [once-a-day] Efficacy (GUIDE) trial, a 6-month double-blind, multicentre trial in Spain and Portugal examining placebo versus GS (1,500 mg once daily) versus acetaminophen (3,000 mg/day), has also recently been presented [8, 27]. The primary efficacy variable was a change in the Lequesne Algo-Functional index. Although there was a numeric difference in improvement in the Lequesne Algo-Functional index between acetaminophen and placebo, only the improvement in the Lequesne Algo-Functional index for GS versus placebo was significant (*p* = 0.032). Secondary analyses, including the OARSI responder indices, were significant for glucosamine (*p* = 0.004).

There are several potential confounders that may have relevance when trying to interpret the seemingly contradictory results of the clinical trials, such as the GAIT and GUIDE.

1. In North America, glucosamine hydrochloride or sulfate and chondroitin sulfate are considered nutraceuticals, whereas in most European countries, these are marketed as pharmaceuticals. Therefore, production and marketing of glucosamine are more closely monitored in Europe. In North America, varying quantities of glucosamine have been noted in a survey of several nutraceuticals [28].
2. Most of the negative clinical trials were performed with glucosamine hydrochloride 500 mg three times daily, whereas most of the positive trials were performed with the GS powder for oral solution at the dose of 1,500 mg once daily. This obviously raises the question, so far unanswered, of the importance of sulfate and of its contribution to the overall effects of glucosamine. Although the sulfate is readily hydrolyzed from the glucosamine in the gastrointestinal tract, there are suggestions that sulfate is in itself clinically relevant [29, 30].
3. Interestingly, the most clinically relevant results in GAIT were seen when sodium chondroitin sulfate was taken with glucosamine hydrochloride; whether this may be explained by an increase in the bioavailability of sulfates together with glucosamine requires further study. It is of note that several of the glucosamine preparations contain other salts that could potentially influence uptake and utilization of glucosamine [31].
4. The placebo response for many clinical trials with oral agents in treatment for knee OA has traditionally been around 30 % [32] and these usual figures were replicated in the GUIDE study. The high placebo response in the GAIT (60.1 %) is of unknown significance.

Although there has been a public comment that the differences in the trials are due to corporate vs noncorporate sponsorship, there have been no data produced to support such allegation. Indeed, one could argue that the differences in results were more from the differences in product, study design, and study populations [33].

The symptomatic efficacy of glucosamine in OA has been analyzed through high-quality quantitative systematic reviews [34–37].

The Cochrane Database of Systematic Reviews on glucosamine included 20 studies with 2,570 patients. Pooled results from studies using a noncrystalline preparation or adequate allocation concealment failed to show benefit in pain and WOMAC function, while those studies evaluating the crystalline preparation show that glucosamine was superior to placebo in the treatment of pain and functional impairment resulting from symptomatic OA. Glucosamine was found to be superior for pain (SMD −1.31, 95 % CI −1.99, −0.64) and function using the Lequesne index (SMD −0.51, 95 % CI −0.96, −0.05). WOMAC outcomes of pain, stiffness, and function did not show a superiority of glucosamine over placebo for both crystalline and noncrystalline preparations of glucosamine. Glucosamine was considered as safe as placebo, in terms of the number of subjects reporting adverse reactions (RR = 0.97, 95 % CI 0.88, 1.08) [38].

Recommendations using on the GRADE (Grading of Recommendations Assessment, Development and Evaluation) system, a system based on a sequential assessment of the quality of evidence, followed by assessment of the balance between benefits versus downsides and subsequent judgment about the strength of recommendations, concluded that glucosamine sulfate demonstrated pain reduction and physical function improvement with very low toxicity and with moderate- to high-quality evidence [39].

## Structural effects in osteoarthritis

To test the long-term effects of GS on the progression of OA joints structural changes and symptoms, two parallel studies including, respectively, 212 and 202 patients with knee OA were designed. Patients were randomly assigned in a double-blind fashion to a continuous treatment with GS (1,500 mg once/day) or placebo for 3 years. Weight-bearing, anteroposterior radiographs of each knee were taken at enrollment and after 1 and 3 years, standardizing patients’ positioning and radiographic procedures. Total mean joint space width of the medial compartment of the tibiofemoral joint was assessed by digital image analysis by a validated computerized algorithm, with the narrowest joint space at enrollment being taken for the primary evaluation (signal joint). Symptoms were scored at each 4-month visit by a total WOMAC index or Lequesne’s Algo-Functional index.

In the first trial, the 106 patients on placebo had progressive joint space narrowing, with a mean joint space loss after 3 years of −0.31 mm (95 % = −0.48 to −0.13). There was no significant joint space loss in the 106 patients on glucosamine sulfate: −0.06 mm (−0.22 to 0.09). Similar results were reported with minimum joint space narrowing. As assessed by WOMAC scores, symptoms worsened slightly in patients on placebo compared with the improvement observed after treatment with glucosamine sulfate. There were no differences in safety or reasons for early withdrawal between the treatment and placebo groups [40].

In the second trial, progressive joint space narrowing with placebo use was −0.19 mm (95 % confidential interval, −0.29 to −0.09 mm) after 3 years. Conversely, there was no average change with glucosamine sulfate use (0.04 mm; 95 % confidence interval, −0.06 to 0.14 mm), with a significant difference between groups (*p* = 0.001). Fewer patients treated with glucosamine sulfate experienced predefined severe narrowing (>0.5 mm): 5 vs. 14 % (*p* = 0.05). Symptoms improved modestly with placebo use but as much as 20–25 % with glucosamine sulfate use, with significant final differences on the Lequesne index and the WOMAC total index and pain, function, and stiffness subscales. Safety was good and without differences between groups [41].

Additional post hoc analyses were performed in order to identify patients who would be particularly responsive to GS as a symptom or structure-modifying drug.

At baseline, in the overall population, mean joint space width (JSW) and narrowest joint space (NJS) point were not significantly correlated with the scores recorded for the WOMAC global index or its pain, stiffness, or function subscales. A statistically significant correlation was observed between the joint space narrowing over 3 years and stiffness or function subscale of the WOMAC during the same period. The 3-year changes in the global WOMAC index in patients within the lowest and highest quartiles of mean joint space width at baseline showed, in both cases, a statistically (*p* < 0.05) significant favorable difference between patients treated with glucosamine sulfate and those having received a placebo [42].

In the placebo group, baseline joint space width was significantly and negatively correlated with the joint space narrowing observed after 3 years (*r* = 0.34, *p* = 0.003). In the lowest quartile of baseline mean joint space width (<4.5 mm), the joint space width increased after 3 years by a mean of 3.8 % (SD: 23.8) in the placebo group and 6.2 % (SD: 17.5) in the glucosamine sulfate group. The difference between the two groups of patients’ with severe OA at baseline was not statistically significant (*p* = 0.70). In the highest quartile of baseline mean joint space width (>6.2 mm), a joint space narrowing of 14.9 % (SD: 17.9) occurred in the placebo group after 3 years, while patients from the glucosamine sulfate group, only experienced a narrowing of 6.0 % (SD: 15.1). Patients with the most severe OA at baseline had a relative risk (RR) of 0.42 (0.17–1.01) to experience a 0.5 mm joint space narrowing over 3 years, compared to those with the less affected joint. In patients with mild OA, (i.e., in the highest quartile of baseline mean joint space width), glucosamine sulfate use was associated with a trend (*p* = 0.10) toward a significant reduction in joint space narrowing [43].

These results were further supported by the demonstration that patients with the highest cartilage turnover at baseline, presented a decrease in collagen type II degradation (CTXII) after 12 months of GS therapy, and that these changes in CTX-II were correlated with the changes in average joint space width observed after 36 months [44].

These results suggest that patients with a less severe radiographic knee OA will be particularly responsive to GS as a structure-modifying drug. However, GS provides long-term relief of symptoms independently of baseline joint space width in patients with mild to moderate osteoarthritis of the knee.

These studies were, however, challenged for the potential systematic error that might have been introduced by the major effect observed—the significant improvement of symptoms in the GS-treated patients compared with placebo-treated patients. It has been hypothesized that the concomitant reduction in pain seen in the glucosamine sulfate arm, relative to placebo, altered the positioning of the knee (in particular favoring a better knee full extension), resulting in a change in joint space width that might have confounded the estimate of joint space narrowing and exaggerated the difference between treatment groups [45]. This hypothesis, however, was demonstrated to be wrong when it was shown that patients from the placebo group, with a major clinical improvement, observed over 3 years, did actually present with a joint space narrowing, while patients with a similar significant symptomatic response, in the GS group, did not experience this structural progression. Patients completing the 3-year treatment course were selected based on a WOMAC pain decrease at least equal to the mean improvement in the glucosamine sulfate arms in either of the original studies, irrespective of treatment with glucosamine sulfate or placebo (drug responders or placebo responders). In a second approach, 3-year completers were selected if their baseline standing knee pain was “severe” or “extreme” and improved by any degree at the end of the trials. In both cases, changes in minimum joint space width were compared between treatment groups. The placebo subsets in both studies underwent an evident mean (SD) joint space narrowing, which was not observed with glucosamine sulfate. Similar results were found in the smaller subsets with greater than or equally severe baseline standing knee pain that improved after 3 years, with a joint space narrowing with placebo not observed with glucosamine sulfate [46].

Although joint space narrowing, as judged on a standardized radiograph, is considered by regulatory agencies as an appropriate primary endpoint for the evaluation of drugs, whether the progression of OA is slowed down through the use of GS has not been unequivocally established [2].

Knee OA patients participating in two above-referenced randomized, placebo-controlled, double-blind, 3-year trials of glucosamine sulfate and receiving treatment for at least 12 months were systematically contacted to participate in a long-term follow-up retrospective assessment of the incidence of total knee replacement.

Out of 340 patients with at least 12 months of treatment, 275 (i.e., 81 %) could be retrieved and interviewed 131 formerly on placebo and 144 on glucosamine sulfate. There were no differences in baseline disease characteristics between groups or with the patients lost to follow-up. The mean duration of follow-up was approximately 5 years after trial termination and treatment discontinuation, making up a total of 2,178 patient-years of observation (including treatment and follow-up).

Total knee replacement had occurred in over twice as many patients from the placebo group, 19 out of 131 (14.5 %), than in those formerly receiving glucosamine sulfate, 9 out of 144 (6.3 %) (*p* = 0.024, chi-square test), with a relative risk that was therefore 0.43 (95 % CI: 0.20–0.92), i.e., a 57 % decrease compared with placebo. The Kaplan–Meier/logrank test survival analysis confirmed a significantly decreased (*p* = 0.026) cumulative incidence of total knee replacements in patients who had received glucosamine sulfate [47].

A 24-month, double-blind, placebo-controlled study, conducted at 9 sites in the United States as part of the glucosamine/chondroitin arthritis intervention trial (GAIT), enrolled 572 patients with knee OA who satisfied radiographic criteria [Kellgren/Lawrence (K/L) grade 2 or grade 3 changes and joint space width (JSW) of at least 2 mm at baseline]. Patients with primarily lateral compartment narrowing at any time point were excluded. Patients who had been randomized to 1 of the 5 groups in the GAIT continued to receive glucosamine HCl 500 mg 3 times daily, CS 400 mg 3 times daily, the combination of glucosamine and CS, celecoxib 200 mg daily, or placebo over 24 months. The minimum medial tibiofemoral JSW was measured at baseline, 12 months, and 24 months. The primary outcome measure was the mean change in JSW from baseline.

The mean JSW loss at 2 years in knees with OA in the placebo group, adjusted for design and clinical factors, was 0.166 mm. No statistically significant difference in mean JSW loss was observed in any treatment group compared with the placebo group. Treatment effects on K/L grade 2 knees, but not on K/L grade 3 knees, showed a trend toward improvement relative to the placebo group. The power of the study was diminished by the limited sample size, variance in JSW measurement, and a smaller than expected loss in JSW [48]. This study confirmed the differences in efficacy between the various glucosamine preparations [49], or doses [50].

In a recent meta-analysis, the authors surveyed randomized, controlled studies that examined the effects of long-term daily glucosamine sulfate and chondroitin sulfate on joint space narrowing (JSN) in knee OA patients using the Medline and the Cochrane Controlled Trials Register, and by performing manual searches. Meta-analysis was performed using a fixed effect model because no between-study heterogeneity was evident. Six studies involving 1,502 cases were included in this meta-analysis, which consisted of two studies on glucosamine sulfate. Glucosamine sulfate did not show a significant effect versus controls on minimum JSN over the first year of treatment (SMD 0.078, 95 % CI −0.116 to −0.273, *p* = 0.429). However, after 3 years of treatment, glucosamine sulfate revealed a small to moderate protective effect on minimum JSN (SMD 0.432, 95 % CI 0.235–0.628, *p* < 0.001). This meta-analysis of available data shows that glucosamine sulfate may delay radiological progression of OA of the knee after daily administration for over 2 or 3 years [51].

## Tolerance

The safety profile of GS was evaluated in a systematic review of 12 randomized, controlled trials and was deemed excellent, with 7 of 1,486 patients randomized to GS who were withdrawn for GS-related toxicity and only 48 having reported any GS-related adverse reactions [37].

Furthermore, an open study carried out by 252 physicians throughout Portugal evaluated the tolerability of GS in 1,208 patients. Patients were given, 500 mg GS orally, 3 times a day, for a mean period of 50.3 days (range: 13–99 days). Most patients (88 %) reported no side effects. In the remaining 12 % of the study population, the reported adverse effects were generally mild and predominantly affected the gastrointestinal tract (e.g., epigastric pain, heartburn, and diarrhea). All the reported complaints were reversible with discontinuation of GS [52]. While some questions were raised regarding the role of glucosamine in glucose metabolism [53] and the possibility of increased insulin resistance, a detailed review of scientific studies performed with GS ruled out this possibility and re-emphasized the safety of short- and long-term use of GS [54].

While, in Europe, GS is regarded as a medication and is thus subject to the usual quality controls, this is not so in Canada and the United States. In Canada, GS is widely available as a nutritional supplement and is not subject to even rudimentary checks on purity. Glucosamine sulfate is very hygroscopic and unstable. Hence, during manufacturing, varying amounts of potassium or sodium chloride are added to improve stability. Because of concerns that the labelling description may not always be valid [14], commercially available capsules or tablets of GS were analyzed in a coughed, blind manner, with a high-performance liquid chromatography system. The amount of free base varied from 41 to 108 % of the mg content stated on the label; the amount of glucosamine varied from 59 to 138 % even when expressed as sulfate [28]. Therefore, the results obtained with one single preparation of GS, registered as a drug in Europe, cannot be extrapolated to the vast majority of OTC preparations sold without the appropriate quality controls. In conclusion, however, there is a high degree of consistency in the literature to consider that when a quality product free of impurities is used, GS has an excellent profile of safety [36, 55, 56] including no induction of glucose intolerance in healthy adults [40, 57].

## Health economics

A study was designed to explore the cost-effectiveness of GS compared with paracetamol and placebo (PBO) in the treatment for knee osteoarthritis, and a 6-month time horizon and a health care perspective were used. The cost and effectiveness data were derived from Western Ontario and McMaster Universities Osteoarthritis Index data of the Glucosamine Unum In Die (once-a-day) [58] Efficacy trial study by Herrero-Beaumont et al. Clinical effectiveness was converted into utility scores to allow for the computation of cost per quality-adjusted life year (QALY). For the three treatment arms, incremental cost-effectiveness ratio was calculated and statistical uncertainty was explored using a bootstrap simulation. In terms of mean utility score at baseline, 3 and 6 months, no statistically significant difference was observed between the three groups. When considering the mean utility score changes from baseline to 3 and 6 months, no difference was observed in the first case but there was a statistically significant difference from baseline to 6 months with a *p* value of 0.047. When comparing GS with paracetamol, the mean baseline incremental cost-effectiveness ratio (ICER) was dominant and the mean ICER after bootstrapping was −1,376 €/QALY indicating dominance (with 79 % probability). When comparing GS with PBO, the mean baseline and after bootstrapping ICER were 3,617.47 and 4,285 €/QALY, respectively. The authors concluded that GS is a highly cost-effective therapy alternative compared with paracetamol and PBO to treat patients diagnosed with primary knee OA.

## Conclusions

GS has shown positive effects on symptomatic and structural outcomes of knee OA. These results should not be extrapolated to other glucosamine salts [hydrochloride or preparations over-the-counter or food supplements] in which no warranty exists about content, pharmacokinetics, and pharmacodynamics of the tablets.
